# Psychological stress in inflammatory bowel disease: Psychoneuroimmunological insights into bidirectional gut–brain communications

**DOI:** 10.3389/fimmu.2022.1016578

**Published:** 2022-10-06

**Authors:** Li Ge, Shuman Liu, Sha Li, Jing Yang, Guangran Hu, Changqing Xu, Wengang Song

**Affiliations:** ^1^ Shandong Provincial Key Laboratory for Rheumatic Disease and Translational Medicine, The First Affiliated Hospital of Shandong First Medical University & Shandong Provincial Qianfoshan Hospital, Jinan, China; ^2^ School of Clinical and Basic Medical Sciences, Shandong First Medical University & Shandong Academy of Medical Sciences, Jinan, China; ^3^ Department of Gastroenterology, The First Affiliated Hospital of Shandong First Medical University & Shandong Provincial Qianfoshan Hospital, Jinan, China

**Keywords:** psychological stress, inflammatory bowel disease, psychoneuroimmunology, brain-gut axis, anxiety, depression, psychiatric comorbidities, psychotherapy

## Abstract

Inflammatory bowel disease (IBD), mainly including ulcerative colitis (UC) and Crohn’s disease (CD), is an autoimmune gastrointestinal disease characterized by chronic inflammation and frequent recurrence. Accumulating evidence has confirmed that chronic psychological stress is considered to trigger IBD deterioration and relapse. Moreover, studies have demonstrated that patients with IBD have a higher risk of developing symptoms of anxiety and depression than healthy individuals. However, the underlying mechanism of the link between psychological stress and IBD remains poorly understood. This review used a psychoneuroimmunology perspective to assess possible neuro-visceral integration, immune modulation, and crucial intestinal microbiome changes in IBD. Furthermore, the bidirectionality of the brain–gut axis was emphasized in the context, indicating that IBD pathophysiology increases the inflammatory response in the central nervous system and further contributes to anxiety- and depression-like behavioral comorbidities. This information will help accurately characterize the link between psychological stress and IBD disease activity. Additionally, the clinical application of functional brain imaging, microbiota-targeted treatment, psychotherapy and antidepressants should be considered during the treatment and diagnosis of IBD with behavioral comorbidities. This review elucidates the significance of more high-quality research combined with large clinical sample sizes and multiple diagnostic methods and psychotherapy, which may help to achieve personalized therapeutic strategies for IBD patients based on stress relief.

## Introduction

Inflammatory bowel disease (IBD), mainly including ulcerative colitis (UC) and Crohn’s disease (CD), is an autoimmune gastrointestinal disease (1). This disease is a common condition affecting approximately 1.5 million people in the United States and 2.2 million people in Europe, with an increasing global incidence (2). Although the exact etiology of IBD remains elusive, the complex interaction between genetic factors, environmental factors, host immune regulation, intestinal microbes and microbial metabolites plays a vital role in the pathogenesis of IBD (3–6). The course of IBD is long and variable, often alternating between periods of quiescent disease and periods of relapsing disease with more active inflammatory episodes (7). Given that IBD is prone to relapse and difficult to treat, it can significantly impact patients’ quality of life (QoL) (8, 9).

In recent years, accumulating evidence has shown that the interaction between brain and gut is closely related to the occurrence and development of gastrointestinal (GI) diseases such as IBD and irritable bowel syndrome (IBS) (10). From this, the concept of brain–gut axis was proposed, which refers to the complex bidirectional communication network between the central nervous system and the intestine (11, 12). This axis enables the cross-talk between the nervous system (including the central nervous system, autonomic nervous system, and enteric nervous system), the endocrine system and the immune system (13–15). A dysregulation of this axis is arguably involved in the pathophysiology of IBD which has long been associated with mental conditions, such as stress, anxiety, and depression. It has been reported that psychological stress is involved in the permeability, motility, sensitivity, and secretion of the intestine, composition of gut microbes, and the promotion of the development and reactivation of intestinal inflammation in animal models of colitis (16–19). Furthermore, in some clinical studies, stress, anxiety, and depression have been considered triggers of IBD relapse and clinical deterioration (20, 21). IBD patients are at higher risk of depression than healthy individuals (22–24). Additionally, a recent meta-analysis has demonstrated that patients with active disease were more prone to experience symptoms of psychological disorders than those with inactive disease (22). Although the bidirectional effects of the brain–gut axis might help explain these observations (25, 26), the complex mechanisms underlying the interaction between psychological stress and the pathophysiology of IBD have not been fully understood.

**Figure 1:**
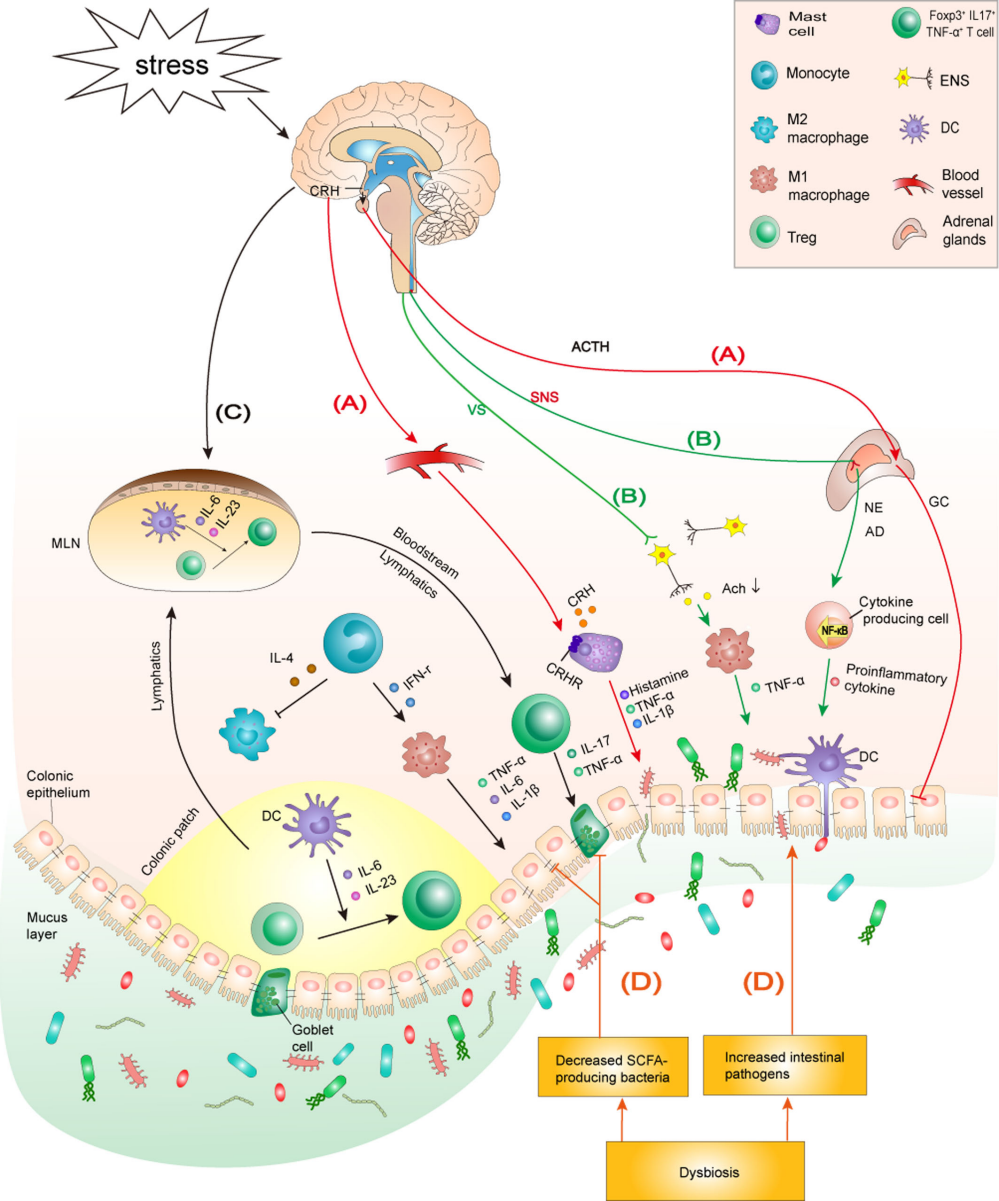
The mechanism by which chronic psychological stress aggravates IBD. **(A)** Stress activates the HPA axis: Stress induces the hypothalamus to release CRH, which triggers the release of ACTH from the hypophysis. ACTH subsequently initiates GC over-synthesis and release in the adrenal cortex. CRH reaches the colon through the blood and binds to CRHRs on the surface of MCs to promote degranulation and the production of proinflammatory cytokines IL-1β and TNF-α, which, in turn, increase the permeability of the colonic epithelial barrier. Furthermore, the overproduction of GC reduces tight junction protein expression between colonocytes, impairing the intestinal barrier function. **(B)** Stress activates the SNS but inhibits the vagus nerve: The sympathetic nervous system is stimulated by stress, which causes the adrenal medulla to secrete excessive AD and NE, promoting the activation of NF-κB signaling and mediating higher secretion of inflammatory cytokines in peripheral tissues. The inhibition of the anti-inflammatory effect of the vagus nerve promotes macrophage-induced TNF-α production, which aggravates the progress of colitis. **(C)** Stress is involved in innate and adaptive immune dysfunction by regulating specific immune cells and cytokine production: Stress induces macrophages to infiltrate the colon and polarize into an M1 phenotype. Additionally, stress promotes intestinal DCs to secrete IL-6 and IL-23, which induces Tregs to differentiate into a Foxp3^+^IL17^+^TNF-α^+^T cell phenotype. M1 macrophages and Foxp3^+^IL17^+^TNF-α^+^T cells secrete corresponding inflammatory cytokines to aggravate the colonic inflammatory response. **(D)** Stress induces microbiome community dysbiosis: Stress causes dysbiosis by increasing intestinal pathogens and decreasing SCFA-producing bacteria in the intestine. Intestinal pathogens can cause and exacerbate colitis by impairing the intestinal barrier and activating intestinal immunity. Decreased SCFAs lead to reduced tight junction protein expression and goblet cell numbers, which in turn lead to impaired intestinal barrier function and aggravated colonic inflammation. HPA axis, hypothalamic-pituitary-adrenal axis; CRH, corticotropin-releasing hormone; ACTH, adrenocorticotropic hormone; GC, glucocorticoids; CRHRs, CRH receptors; ANS, autonomic nervous system; SNS, sympathetic nervous system; AD, adrenaline; NE, noradrenaline; VS, vagus nerve; MCs, mast cells; SCFAs: short-chain fatty acids; MLN, mesenteric lymph node; ENS, enteric nervous system.

**Figure 2:**
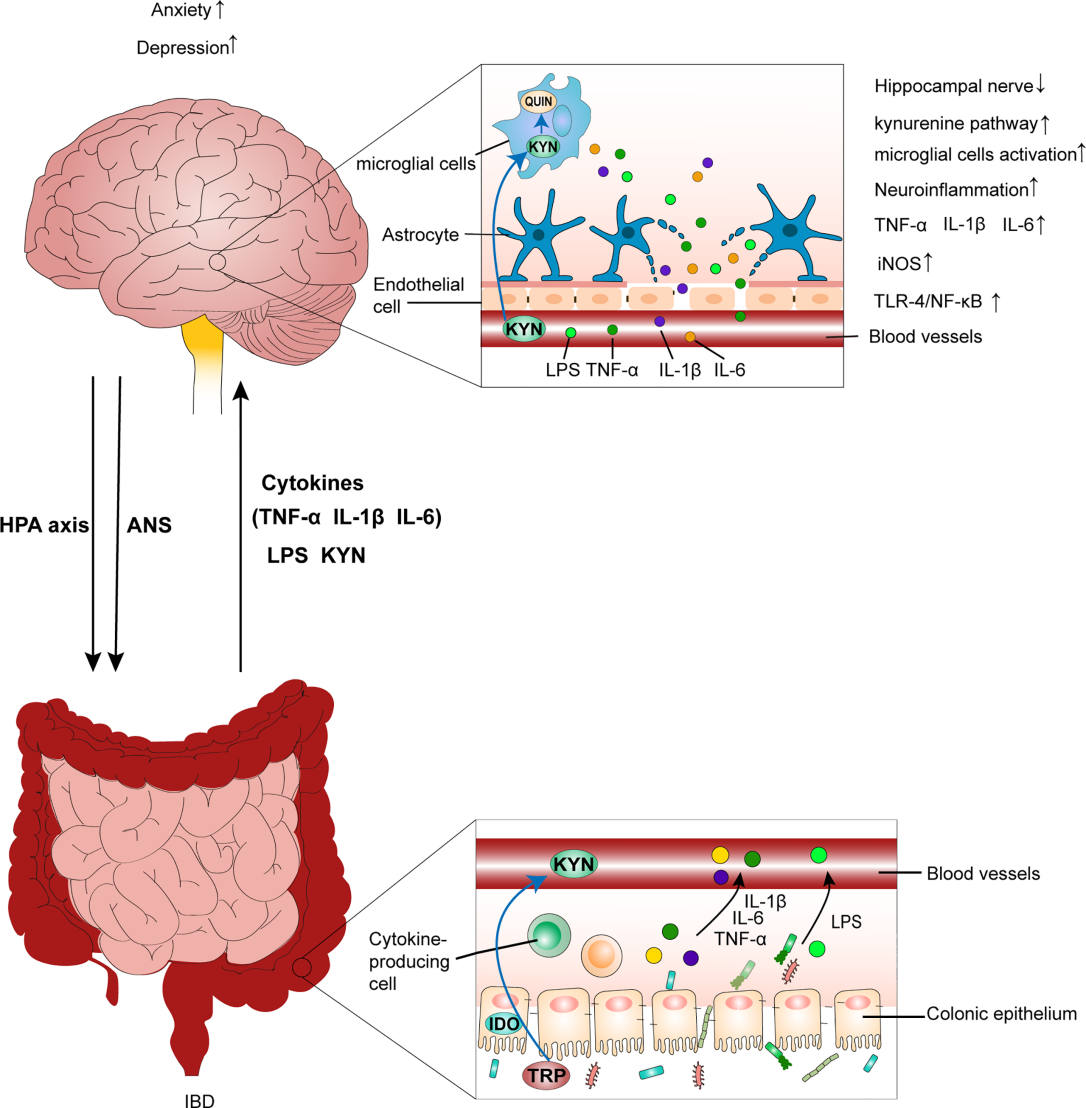
IBD leads to anxiety- and depression-like behavioral comorbidities by inducing neuroinflammation. Chronic psychological stress, such as anxiety and depression, aggravates colitis by activating the HPA axis and disrupting the ANS. This is accompanied by an increase in the production of LPS, pro-inflammatory cytokines (TNF-α, IL-1β, and IL-6), and gut leak. Additionally, peripheral inflammation causes a decrease in tight junction protein expression between endothelial cells in the BBB. Then, the disruption of BBB integrity predisposes the translocation of circulating inflammatory mediators into brain tissues, which leads to the inflammatory response in the CNS by increasing IL-1β, IL-6, and TNF-α levels, activating the TLR4/NF-κB inflammatory pathway, as well as increasing iNOS and nitrite levels in brain tissues. Further, circulating inflammatory mediators reaching the brain affect neuroglial networks and activate microglial cells, which further exacerbate the inflammatory response in the CNS. Additionally, activated microglial cells can promote neurodegeneration of the hippocampal nerve, which also contributes to mood disorders. Furthermore, overactivation of the kynurenine pathway generates excess QUIN in the brain, which impairs neurons and neuroglial cells, resulting in inflammation–mediated depression. IBD, inflammatory bowel disease; HPA axis, hypothalamic-pituitary-adrenal axis; ANS, autonomic nervous system; LPS, lipopolysaccharide; BBB, blood–brain barrier; CNS, central nervous system; TRP, tryptophan; KYN, kynurenine; QUIN, neurotoxic quinolinic acid.

A recent study suggested that stress resulting from the spread of the new coronavirus disease 2019 (COVID-19) has a strikingly positive relationship with CD activity (27). Therefore, in the context of the ongoing epidemic and spread of COVID-19, to elucidate the interplay between mental states and IBD evolution and better understand the bi-directional modulation through the brain–gut axis, it is essential to explore the exact mechanism of the link between mental health and IBD evolution and provide leads for therapeutic interventions. In this review, we used a psychoneuroimmunology perspective to discuss the mechanism by which chronic psychological stress impacts neuroendocrine immune regulation, damages the intestinal immune function and microbiota homeostasis, and subsequently aggravates IBD progression (**Figure 1**). Moreover, we found that disordered gut homeostasis in IBD was responsible for driving the brain pathology, exacerbating inflammatory response in the CNS, and contributing to anxiety- and depression-like behavior (**Figure 2**). Additionally, the application of neuroimaging studies in evaluating possible neuromechanisms involved in IBD and adjunctive effects of some promising microbiota-targeted treatment, psychological therapies and antidepressants are discussed.

## Neuroendocrine stress response pathways

Different regions of the brain, especially the hypothalamus, amygdala, and hippocampus, interact with each other through complex neural networks involved in the generation of stress responses. Stress-induced neuroendocrine changes occur by two effector pathways: the hypothalamic-pituitary-adrenal (HPA) axis and the autonomic nervous system (ANS) (28). The HPA axis is activated by various stressors and, in conjunction with the ANS, enables the body to mount coordinated responses to perceived threats.

### Psychological stress and the HPA axis

The HPA axis is a classic neuroendocrine regulatory network involved in the adaptive response to psychological stress. Chronic psychological stress can induce the lasting activation of the HPA axis (29). The hypothalamus secrets excessive corticotropin-releasing hormone (CRH), triggering the release of adrenocorticotropic hormone (ACTH) from the anterior pituitary gland. ACTH itself stimulates the secretion of adrenal glucocorticoids (GCs) by the zona fasciculata of the adrenal cortex, such as cortisol in humans and corticosterone in rodents (28, 30, 31).

Recent studies have shown that the HPA axis plays a significant role in stress and intestinal diseases. Firstly, CRH might be involved in the regulation of the intestinal barrier function, intestinal peristalsis, and secretion function in some intestinal diseases, such as IBS and IBD (32). Some studies have indicated that corticotropin-releasing hormone receptors (CRHRs) on the surface of mast cells (MCs), dendritic cells (DCs), T cells, and other gastrointestinal immune cells bind to excessive CRH released by the hypothalamus during stress responses, inducing MC degranulation, T cell-related adaptive immune response activation, increased intestinal-epithelial permeability, and increased pro-inflammatory cytokine production (33–36). In a recent study conducted on humans, the stress induced by public speaking raised serum cortisol levels in some subjects, and it was only in these subjects that the intestinal barrier function was disrupted, suggesting that the HPA axis was activated. Moreover, exogenous CRH administration in humans reproduced the impact of public speaking-induced psychological stress, which increased small intestine permeability (37). Additionally, the overproduction of GCs and CRH induced by chronic psychological stress reduces the expression of tight junctions in the intestine, damaging the function of the intestinal mucosal barrier, which is closely related to IBD progression (38–40).

### Psychological stress and the ANS

Stress controls the autonomic response by stimulating the sympathetic nervous system, which causes the adrenal medulla to secrete excess epinephrine and norepinephrine, subsequently inducing central and peripheral tissues to secrete excessive inflammatory mediators/proinflammatory cytokines and promote the activation of inflammatory nuclear factor-κB (NF-κB) signaling (41). The gut itself has a rich supply of nerves, called the enteric nervous system (ENS). The neurons of the sympathetic and parasympathetic nervous system communicate with enteric nerves to form a complex neural network that has been termed the brain–gut axis. The ENS contains a large number of neurons that are responsible for regulating many functions of the gastrointestinal tract (28, 42). The stress-induced activation of the sympathetic nervous system excites the enteric ENS and increases the density of nerve fibers and the number of cholinergic neurons in the intestinal mucosa, leading to higher permeability of the intestinal epithelium (43, 44). Clinical studies have shown that lesions in CD and UC patients have a higher number of submucosal nervous plexuses and increased epithelial permeability (45).

The vagus nerve is a parasympathetic nerve which can achieve bidirectional communication between the CNS and the gastrointestinal tract (46). The release of acetylcholine from the efferent fibers of the vagus nerve inhibits macrophage-induced tumor necrosis factor (TNF)-α production through interneurons of ENS synapses between efferent vagus nerve endings and macrophages. Secretions from the vagus nerve are released through synapses and can also inhibit TNF-α secretion by splenic macrophages (46). Given the impact of TNF-α on IBD progression, the vagal inhibition of TNF-α secretion could be a promising avenue for treatment (47–49). A newly-identified vasovagal liver–brain–gut reflex circuit could control the number of peripheral regulatory T cells and maintain gut homeostasis (50).

It has shown that psychological stress inhibits the anti-inflammatory effect of the vagus nerve as stress reduces nerve tension and accelerates the production of inflammatory cytokines in several diseases, such as treatment-refractory depression, posttraumatic stress disorder, and IBD (46, 51, 52). Meta-analyses have indicated that the expression levels of some proinflammatory cytokines, like interleukin (IL)-1, IL-6, and TNF-α are significantly upregulated during depressive episodes (53, 54). In both UC and CD patients, lower vagal activity was associated with systemic inflammatory manifestations, especially excessive TNF-α and C-reactive protein (CRP) production (49, 55). Systemic inflammation, by itself, can lead to a depressive state and promote IBD flare-ups (20, 56, 57). Excitedly, vagus nerve stimulation (VNS) has shown promise for modulating neuroinflammation and suppressing intestinal inflammation associated with IBD (58–63). Kibleur et al. (60) have found that chronic VNS decreased anxiety and resulted in general clinical improvement of CD. An additional study demonstrated that VNS improved the intestinal barrier function by promoting intestinal epithelial renewal (48). Thus, by restoring vagus nerve activity, VNS is potentially useful in the treatment of IBD (46).

## Chronic psychological stress disturbs intestinal immune function during IBD

The dysfunction of innate and adaptive immune pathways is the primary cause of intestinal inflammation in patients with IBD (10, 64, 65). It is well known that the immunopathogenesis of IBD has been involved in mediation by various different cell types, including classic immune cells, like macrophages, MCs, neutrophils, DCs, monocytes, and adaptive immune cells, such as T cells, and nonimmune cells, like epithelial cells, endothelial cells, and mesenchymal cells (66, 67). However, it is still unclear to what extent psychological stress is involved in the regulation of the immunopathology of these immune cells during the aggravation/relapse of IBD or intestinal inflammation. In other words, spatiotemporal regulation of psychological stress on these immune cells and cytokine production is important to elucidate the mechanism underlying stress-mediated IBD/intestinal inflammation. **Table 1** presents a summary of the stress-induced dysfunction of specific immune cells and the mechanisms involved in IBD progression/inflammatory aggravation.

**Table 1:** Stress-induced dysfunction of specific immune cells and mechanisms promoting IBD development/inflammatory aggravation.

Cell type	Species	Stress model	Stress-induced cell dysfunction	Mechanisms/Conclusions
MCs	Rat	CWAS	MC hyperplasia and increased activation in the mucosa.	MC-dependent ultrastructural damage in epithelial cells, decreased the number of goblet cells, adherence and internalization of bacteria in the epithelium, and inflammatory cell infiltration in the lamina propria, which induced barrier dysfunction of the ileum and colon. None of these changes occurred in MC-deficient rats (68).
MCs	Human	Public speech stress, Intraperitoneal injection of CRH	MCs were activated and released proteases and inflammatory cytokines, such as TNF- α.	Acute public speech stress mediates the increase in CRH on the HPA axis. CRH binds to CRH1R on human intestinal mucosal MC, activates MC, and releases protease and proinflammatory cytokines, such as TNF-α, increasing intestinal permeability (37).
MCs	Mice	ARS	MC hyperactivity with female predominance. Female mice had a higher capacity for MC mediator synthesis, storage, and release than males.	Estrogen promotes stress-induced mast cell degranulation through GPER. Thus, female mice exhibited a greater serum histamine response and intestinal permeability than male ones, however, there were no differences in corticosterone secretion (69, 70).
MCs	IL10^-^/^-^ Mice	NMS	Increased MC activation and colonic tryptase release.	Increased MC activation and colonic tryptase release damage the expression of tight junction protein between colonocytes and promote neutrophil recruitment and subsequent reactivate the immune response (71).
Mac	Mice	SDR	Increases in colonic F4/80^+^Mac.	SDR exposure leads to increased production of CCL2 in the colon. CCL2 recruits inflammatory monocytes from the bone marrow into the colon and differentiate into Mac. Increased Mac produces excessive inflammatory cytokines and mediators, such as TNF-α and iNOS, which exacerbate *Citrobacter rodentium*-induced colitis (72).
Mac	Mice	Intraperitoneal injected CRH	Enhanced M1/M2 polarization in the left colon from IBD mice.	Peripheral administration of CRH increased M1/M2 polarization, aggravating intestinal inflammation in IBD mice. Blocking intestinal autophagy significantly attenuated this effect (73).
Mac	Human	Depression	Monocyte subpopulation disequilibrium toward intermediate and non-classic phenotypes and Mac polarization towards an M1 phenotype.	M1 macrophages strongly express iNOS and pro-inflammatory cytokines, such as IL-1β, IL-6, and TNF-α, deteriorating intestinal inflammation (74).
Mac	Mice	CSDS	Induce autophagy-dependent degradation of NLRP3 in Mac.	CSDS induced a marked accumulation of kynurenic acid in the intestine; the kynurenic acid/GPR35 axis promotes autophagy-mediated NLRP3 degradation in macrophages, which aggravates colitis injury. Blocking intestinal autophagy partially reversed this effect (75).
Th17/Treg	Mice	CRS	The inhibitory ability of intestinal Treg cells was compromised while Treg cell frequency in the intestine remained unchanged. CRS induced Foxp3^+^ Treg cells to differentiate into Foxp3^+^ IL17^+^ TNF-α^+^ T cells in the intestine.	CRS-derived prolactin increases the production of IL-6 and IL-23 by activating NF-κB signaling in DCs. IL-6 and IL-23 induced Treg cells to lose immunosuppressive function and express high levels of TNF-α (76).
Th17/Treg	Mice	CSDS	Decreased splenic T cell frequency, increased IL-17-producing CD4^+^ and CD8^+^ T cells, and reduced Treg numbers and TGF-β gene expression in the spleens of susceptible mice.	CSDS promoted Th17 cell differentiation and suppressed Treg cell differentiation, and increased the levels of serum IL-1β and IL-6 predominantly in susceptible mice (77).
Th17/Treg	Mice	CUMS	Upregulated the ratio of Th17/Treg.	CUMS promoted the infiltration of immune cells and increased the levels of pro-inflammatory cytokines IL-17A and IL-1β in ileum, enhancing the severity of intestinal inflammation (78, 79).
Neutrophil	Mice	CRS	Promotes neutrophil infiltration in colon tissue.	CRS resulted in the elevated expression of pro-inflammatory cytokines (IL-1β, IL-6, IL-17A, and IL-22) and neutrophil chemokines (CXCL1 and CXCL2), and promoted neutrophil infiltration into colonic tissues (80).

MCs, mast cells; Mac, macrophage; Th17, T helper type-17 cell; Treg, regulatory T cell; CRS, chronic restraint stress; CWAS, chronic repeated water avoidance stress; CRH, corticotropin-releasing hormone; ARS, acute restraint stress; GPER, G-protein-coupled estrogen receptor; NMS, neonatal maternal separation; SDR, social disruption; CSDS, chronic social defeat stress; CUMS, chronic unpredictable mild stress.

### Chronic psychological stress activates intestinal mucosal MCs

MCs are effectors of the brain–gut axis that produce neurotransmitters and pro-inflammatory cytokines in response to stress signals to regulate the permeability of the intestinal epithelial barrier *via* the HPA axis (81). Animal experiments have shown that chronic psychological stress-induced CRH is released by the hypothalamus and binds to MC CRHR-1 to induce activation and degranulation of MC and increase the permeability of the intestinal epithelium while blocking CRH release inhibits MC degranulation and the production of TNF-α and protease, which delays the impairment of intestinal barrier. Intriguingly, in stressed rats with an MC defect, intestinal epithelial function and morphology were not markedly altered (68, 82). One Clinical study found that public speaking-caused psychological stress results in the activation of HPA axis and the elevated level of CRH only in some volunteers that the intestinal permeability was higher than the other volunteers, whereas, symptoms could be eliminated by MC stabilizers (37). These studies have implied that psychological stress aggravates the permeability of the intestinal epithelial barrier likely through an intestinal mucosal MC activation-dependent pathway (37). Additionally, the study of Mackey et al. (69) found that when C57BL/6 mice were exposed to restraint stress (RS), female mice had significantly higher intestinal permeability, serum histamine levels, and MC degranulation than male ones, suggesting that changes to the intestinal epithelial barrier induced by psychological stress through the regulation of MCs were sex-specific. This phenomenon might be caused by G-protein-coupled estrogen receptor (GPER) on MCs of the colonic mucosa. Estrogen promotes stress-induced MC degranulation through GPER (70). In a comorbid mice model of neonatal maternal separation (NMS) stress and colitis, the researchers have explored that NMS stress exacerbates colitis in IL10^-^/^-^ mice by disrupting intestinal barrier. Notably, increased MC activation and colonic tryptase release were found which could damage the expression of tight junction protein between colonocytes and promote neutrophil recruitment and subsequent reactivate the immune response, thus like play a vital role in defects in intestinal barrier function (71).

### Chronic psychological stress promotes M1 phenotype polarization and autophagy in macrophages

Macrophages play a key role in IBD pathogenesis, and macrophage infiltration, along with an imbalance in the macrophage M1/M2 ratio, is associated with IBD development (83, 84). Recent studies have indicated that macrophages are involved in the pathophysiology of psychological stress-induced IBD aggravation. Social disruption (SDR) stress has been shown to increase the number of F4/80^+^ macrophages in the colon and thus enhanced severity of infectious colitis in mice (72). Additionally, as the stress mediator, CRH can abnormally enhance the macrophage M1/M2 polarization in the left colon of mice with IBD (73). Similarly, in clinical studies, depression promoted the migration of monocytes from the blood to the intestinal mucosa, causing macrophages to infiltrate colonic tissues, which aggravates inflammation. CD patients with depression have a higher number of M1 macrophages and higher levels of their secreted proinflammatory cytokines and a lower number of M2 macrophages and lower levels of their secreted anti-inflammatory cytokines (e.g., IL-10) than those without depression, suggesting that depression promotes macrophage polarization into an M1 phenotype and accelerates IBD progression (74). Another recent study has shown that the stress-induced over-activation of macrophage autophagy might also aggravate IBD (75). The results indicated that chronic social defeat stress (CSDS) caused kynurenic acid accumulation in mouse colons, which, in turn, enhanced macrophage autophagy in the intestine. This induced the defective activation of NLRP3 inflammasomes, making mice more vulnerable to dextran sulfate sodium (DSS)-induced colitis (75). Thus, it is possible that chronic psychological stress is involved in IBD’s pathophysiology by promoting the infiltration of macrophages into the colon and polarization of macrophages into an M1 phenotype, which both enhance autophagy.

### Chronic psychological stress regulates T cell differentiation, phenotype, and function

CD4^+^CD25^+^Foxp3^+^cells, which express the inhibitory receptor, CTLA-4, and produce high levels of IL-10 and TGF-β, are the key regulatory T cells (Tregs) responsible for inducing immune tolerance and preventing colitis by inhibiting immune effector cell activation through cell contact and inhibitory cytokine production (76, 85). The unbalanced T helper type-17 (Th17) cell/Treg ratio associated with intestinal inflammation is a typical feature of the disruption in mucosal immune homeostasis (86–88). Present studies indicate psychological stress-induced alterations in the differentiation frequency of Th17 and Treg subsets. In CSDS mouse model, stress enhanced splenic IL-17-producing Th17 differentiation, suppressed Treg cell differentiation and decreased the levels of TGF-β in the stress susceptible mice (77). Similarly, in CUMS mouse models, stress upregulated the ratio of Th17/Treg in the ileum, disrupted immune homeostasis by activating the inflammatory response and thereby enhance intestinal inflammation (78, 79). These studies have implied that an investigation of the precise mechanism by which chronic psychological stress impacts Th17 and Treg subset differentiation is imperative to understand the mechanism of stress-induced adaptive immunity response of intestinal T cells.

A recent study found that chronic psychological stress also induced phenotypic and functional changes in T cell subsets. In CRS mouse models, chronic stress did not change the frequency of Treg differentiation in the intestine but could induce phenotypic changes in Treg cells and damage their inhibitory function. In the study, chronic psychological stress stimulated serum prolactin secretion and, in turn, activated inflammatory NF-κB signaling to induce IL-6 and IL-23 production from DCs. This change in the intestinal DC phenotype induced Tregs to differentiate into Foxp3^+^ IL17^+^ TNF-α^+^ T cells that produced a lot of TNF-α and enhanced intestinal inflammation. The study also showed that chronic stress aggravated colitis symptoms in mice on DSS/TNBS treatment (76). These findings showed that chronic psychological stress regulates the differentiation frequency, phenotype, and function of Th17 and/or Treg cells in a complex manner, providing important research directions for the study of how chronic psychological stress regulates intestinal immune function and affects IBD development.

Except for the immune cells mentioned above, neutrophils may also be one of the target cells of psychological stress-induced IBD. A recent study revealed that chronic psychological stress upregulated the levels of neutrophil chemokines (CXCL1 and CXCL2) which stimulated neutrophil mobilization and infiltration into colonic tissues, thereby secreted excessive proinflammatory cytokines (IL-1β, IL-6, IL-17A, and IL-22), so the robust inflammatory response aggravating colitis in a DSS-induced mice model (80). Obviously, there are many other immune cells involved in the occurrence of IBD, such as natural killer (NK) cells, natural killer T (NKT) cells, and innate lymphoid cells (ILCs) (67). However, whether these immune cells are involved in the mechanisms underlying the interaction of psychological disorders with the IBD progression has not been studied. Perhaps, such studies should be initiated under both experimental conditions and human clinical trials.

### Neuroimmune modulation of biogenic amines with focus on Th17 Cells

It has shown that biogenic amines, mainly including catecholaminergic dopamine, norepinephrine, and adrenaline, 5-hydroxytryptamine (5-HT)/serotonin, and histamine, are direct neurotransmitters present in central and peripheral tissues with potential effects on neuroimmune interaction and brain–gut axis (89–92). Particularly, serotonin is one of the critical neurotransmitters that not only involves the pathogenesis of various psychological and psychotic diseases, but also alleviates neuroinflammation by manipulating immune cell activity and cytokine production (93). More than 90% of serotonin is contained in the gut and accumulating evidence has demonstrated that serotonin may be of relevance in relation to the psychopathology of IBD and IBD with psychological comorbidities by modulating the IL-17/Th17 signaling response (89, 90). It has demonstrated that serotonin follows a receptor-specific pattern to suppress the release of inflammatory mediators IL-1β, IL-6, TGF-β3 and IL-23 and thus inhibit the development of pathogenic Th17 cells (94–96).

Along with biogenic amines, recent studies have shown the critical pathogenetic role of Th17-cells and Th17-immune response in participation in the gut-brain axis to mediate chronic neuroinflammatory and autoimmune diseases (97). Intriguingly, studies have found that compared to that of healthy control, patients with depression have decreased Tregs and concentration of serotonin, as well as increased Th17 cells (92, 98). Th17-cells produce pro-inflammatory cytokines such as IL-17A, IL-21, IL-22, and interferon-γ (IFN-γ) (99, 100). IL-17A production and the expression of IL-6 and chemokine receptor-6 promote penetration of Th17-cells through the blood–brain barrier (BBB) into the CNS and induced the mental disorders and neuroinflammation (101, 102).

Although the precise mechanisms of action of Th17 cells in depression remain unclear, Th17 cells appear as a promising therapeutic target for depression. The neutralization of IL-17A by anti-IL-17A antibodies or supplement of serotonin to block the release of gut Th17 cells might represent a reasonable and feasible therapeutic approaches to improve depression symptoms (92).

In addition to regulating immune cells directly, psychological stress can also affect host immunity through altering the composition and abundance of the intestinal microbiota. The disturbed intestinal microbiota induced the increased expression of inflammatory cytokines both in the gut and in systemic tissues by promoting the colonization and amplification of pathogens, activating immune responses, and promoting intestinal inflammation (103, 104). Therefore, the precise mechanism by which stress manipulates intestinal microbiota and thus shapes host immunity has become a central segment to further analyzing the pathology of IBD with psychological comorbidities.

## Chronic psychological stress disrupts the intestinal microbiome in IBD

The brain–gut axis is a sophisticated bidirectional communication network between the CNS and the intestine. Recently, the intestinal microbiome has been regarded as the third critical component of the brain–gut axis, and the concept of a brain–gut–microbiota axis has been proposed (105, 106). As mentioned above, psychological stressors can change to the composition of intestinal microbiota, and promoting intestinal inflammation (103, 104). In this context, increasing attention has been placed on the interactive regulatory mechanism of the brain and microbiota during disease progression (107, 108). **Table 2** provides a summary of stress-induced microbiome community dysbiosis and the mechanisms involved in IBD progression/inflammatory aggravation.

**Table 2:** Stress-induced microbiota dysbiosis and mechanism promoting IBD/inflammatory aggravation.

Species	Stress model	Stress-induced microbiota dysbiosis	Mechanism/Conclusions
Mice	6 days of short-term SDR	Decreased the relative abundance of *Bacteroides*, *Coprococcus* spp.*, Dorea* spp., and *Pseudobutyrivibrio* spp. while increasing the relative abundance of *Clostridium*.	Increased levels of circulating IL-6 and monocyte chemoattractant protein-1 were directly related to a decrease in microbiome abundance, including *Coprococcus* spp.*, Dorea* spp., and *Pseudobutyrivibrio* spp., which could allow for an increase in the abundance of *Clostridium*, and, subsequently, translocated and induced an inflammatory response (104).
Mice	7 days of short-term RS	Microbial richness and diversity decreased. After the oral administration of *C. rodentium*, the colonization of *C. rodentium* increased significantly.	The increased colonization of the *C. rodentium* pathogen triggers an increase in TNF-α and iNOS gene expression in colonic tissues and causes severe intestinal inflammation (109).
Rat	Cold stress	A significant increase in Proteobacteria.	The increase in the abundance of pro-inflammatory Proteobacteria and the upregulation for the levels of pro-inflammatory cytokines and biomarkers, such as IL-1β, TNF-α, and Cox-2, in the colonic mucosa (110).
Mice	Immobilization stress	Increased the relative abundance of Proteobacteria and reduced the relative abundance of Firmicutes and Actinobacteria in the fecal microbiota.	The immobilization stress (IS) induced the increase of fecal Proteobacteria population and overproduction of LPS, led to gut immune responses, and accelerated the development of colitis (111).
Mice	Immobilization stress	Increased Proteobacteria, and Firmicutes populations and decreased the Bacteroidetes population in the fecal samples.	The IS induced disturbance of the gut microbiota composition, particularly increased the pathogenic Proteobacteria population. Transplanting the feces of mice exposed to IS into normal mice could induce colonic shortening, increase myeloperoxidase activity, the expression of IL-1β, TNF-α, and IL-6, and NF-κB^+/^CD11c^+^ cell population in the colon (112).
Mice	CRS	Increased relative abundance of inflammation-related bacteria, including *Helicobacte*r, Peptostreptococcaceae, *Streptococcus*, and *Enterococcus faecalis*.	Increased abundance of specific inflammation-related bacteria activated the IL-6/STAT3 signaling pathway, which facilitated the development of DSS-induced colitis. This was abolished after antibiotic treatment (17).
Mice	CSC	CSC increased the relative abundance of *Helicobacter* and *Paraprevotella.*	CSC exposure activated the host immune response toward *Helicobacter* and *Paraprevotella*, increased the number of mesenteric lymph node cells, and promoted the release of IFN-γ and IL-6, which induced the progress of spontaneous colitis or the deterioration of experimental colitis (113).
Mice	CWAS	In B6-Tcra^−/−^ mice, exposure to CWAS increased the level of the genus *Clostridium*.	Increased relative abundance of the genus Clostridium induced the increase in production of the toxin, phospholipase C, which aggravate the colitis severity (114, 115).
Mice and Human	CRS	In mice under CRS and UC with depression, the abundance of *Akkermansia muciniphila* was significantly decreased.	The decreased abundance of *Akkermansia muciniphila* accelerated the microbial-mediated disruption of intestinal barrier function and colitis aggravation (16).
Mice	PNMS	An increase in *Desulfovibrio*, *Streptococcus*, and *Enterococcus* abundance and a decrease in *Bifidobacterium* and *Blautia* abundance in 3-week-old PNMS offspring. The sustained proliferation of *Desulfovibrio* appeared from the weaning period to adulthood.	PNMS inhibited the intestinal development of offspring by increasing the abundance of pathogenic bacteria, especially causing the sustained excessive proliferation of *Desulfovibrio* in the offspring, and eventually led to the deterioration of colonic inflammation in adulthood (116).
Human	PNMS	Infants had significantly higher relative abundances of Proteobacterial groups and lower relative abundances of lactic acid bacteria and Bifidobacteria.	Proteobacterial groups contain pathogens, such as *Escherichia* and *Enterobacter*. This aberrant colonization was related to infant gastrointestinal symptoms and increased levels of inflammation in the gut (117).
Human/Mice	Depression and anxiety	Lower fecal microbial community richness and diversity, with more *Lactobacillales, Sellimonas, Streptococcus*, and *Enterococcus* but less *Prevotella-9* and *Lachnospira.*	Depression/anxiety increased *Streptococcus* and *Enterococcus* but decreased *Prevotella-9* and *Lachnospira*. This disorganized gut microbiota caused immune activation and elevated intestinal TNF-α, IL-6, and LPS levels (118).
Human/Mice	Depression	Depression increased the Enterococcaceae population in IBD patients.	Depression increased the Enterococcaceae population, NF-κB^+^/Iba1^+^ cells, expression of IL-1β and IL-6, in IBD patients, which cause more severe colitis with a disrupted intestinal barrier, and accelerated the translocation of fecal LPS into the blood (119).
Rat	CUMS	CUMS exposure decreased *Prevotella-9* abundance and the fecal SCFA level.	Reductions in the number of SCFA-producing bacteria, *Prevotella-9*, decreased goblet cell numbers, and reduced level of the tight junction protein, occludin 1, which disrupted the mucosal barrier integrity and augmented the expression of inflammatory cytokines, IL-6 and IFN-γ, further exacerbating intestinal inflammation (120).

RS, restraint stress; *C. rodentium*, Citrobacter rodentium; CSC, chronic subordinate colony housing; PNMS, prenatal maternal stress; LPS, lipopolysaccharide; SCFAs, short-chain fatty acids.

A acute stress can reduce the abundance and diversity of bacterial species, increase colonic colonization/amplification of pathogens–, especially the Proteobacteria population, including *Citrobacter rodentium*, *Helicobacter pylori*, and *Clostridium*, and upregulate the expression of inflammatory cytokines in the colon and plasma, this exacerbating gastrointestinal inflammation (104, 109–112). Long-term chronic stimulation also changes the composition of intestinal microbiota, further damaging immune homeostasis in the intestinal mucosa and aggravating colitis (17, 113–115). Chronic stress induced spontaneous colitis or exacerbated chemically-induced colitis by promoting the expansion of pathogens and activating the mucosal immune response (17, 113–115). For example, in chronic subordinate colony housing (CSC) pretreated mice, the abundance of *Helicobacter* and *Paraprevotella* was remarkably increased and simultaneously activated the host’s immune response. After adding DSS, mouse mesenteric lymph node cells were further increased, more IFN-γ and IL-6 was produced, and intestinal tissue damage worsened (113). Similarly, male C57BL/6 mice that received CRS pretreatment and subsequent DSS intervention showed a more significantly increased abundance of pro-inflammatory bacteria, such as Peptostreptococcaceae, *Helicobacter*, *Streptococcus*, and *Enterococcus faecalis*, further increasing inflammation, activating the IL-6/STAT3 inflammatory signaling, and aggravating colitis. Unexpectedly, the stress sensitization of colitis was not stopped in IL-6 knockout mouse models, which suggestes that a high inflammatory response was not the main cause of the chronic stress-aggravated colitis. Conversely, antibiotic treatment eliminated the intestinal microbiota factor and the difference between the stressed group and the non-stressed group (17). Additionally, *Clostridium perfringens* and *Clostridium sordellii* are both pathogens producing phospholipase C, a major virulence mediator. The abundance of these bacteria was increased in water avoidance stress (WAS)-treated mice, and both strains of bacteria have been associated with colitis exacerbation (114, 115). These findings indicated that the key target of chronic stress-induced intestinal inflammation is intestinal microbiota (17, 113–115).

Researchers have also found that the change in microbial flora induced by CRS results in insufficient intestinal mucosal barrier-related protein, mucin-2, thus, disrupting the colonic mucus and aggravating colitis in murine models, suggesting that the microbial flora factor plays a synergetic role in damaging the intestinal barrier (16, 17). More especially, researchers have demonstrated that the population of *Akkermansia muciniphila* (*A. muciniphila*) was obviously reduced in CRS mice and UC patients with depressive symptoms. Remarkably, its abundance changes were positively correlated with mucin-2 expression. The administration of *A. muciniphila* could repair colonic mucus layer and modify the gut microbiota. Their results suggest that *A. muciniphila* plays a beneficial role in protecting the intestinal mucosa in IBD patients with psychological disorders (16). Interestingly, maternal stress during pregnancy also affects the offspring. Prenatal maternal stress (PNMS) can increase the abundance of pathogenic bacteria, especially proteobacterial groups, and decrease the abundance of beneficial bacteria, especially *Bifidobacterium* and *Blautia*, which has profound pathological impacts on the infant’s immune system and ultimately increases the susceptibility of adult mice offspring to colitis (116, 117).

While much of the current understanding of the link between stress and intestinal microbiome comes from animal models (121, 122), many human and patient-related studies of the brain–gut–microbiome axis are in progress (123–125). A few clinical studies have also associated stress with microbiome homeostasis and gastrointestinal health (118, 126, 127). Studies have shown that the richness and diversity of the fecal microbiome were dramatically reduced in IBD patients with anxiety or depression compared to patients without anxiety or depression (118, 126, 127). For patients with IBD in remission, Humbel et al. found that psychological disorders were negatively correlated with the abundance of *Clostridia*, Bacilli, Bacteroidia, and β- and γ-Proteobacteria. Although Proteobacteria is widely considered as pathogenic bacteria, the population of β- and γ-Proteobacteria was generally reduced with increasing anxiety in this study. Thus, the clinical trials with large samples are needed to further confirm the validation of this questionable finding. Psychological disorders have also been linked to a decrease in the composition and richness of some bacteria such as Lachnospiraceae, Fusobacteriaceae, Ruminococcaceae, and Veillonellaceae (127). Additionally, studies have demonstrated that the relative abundance of *Desulfovibrio* in patients with UC and *Bifidobacterium* in patients with CD is associated with depression, whereas the abundance of *Sutterella*, RF 32, and *Lactococcus* associated with patients’ QoL only among patients with CD (127). For patients with active UC, a prospective study of 240 Chinese patients has demonstrated that, in total, almost 50% of the patients had symptoms of anxiety or depression (118). The researchers have found that patients with UC and depression/anxiety (UCD/UCA) had more Lactobacillales, Sellimonas, *Streptococcus*, and *Enterococcus* but less *Prevotella* 9 and *Lachnospira* than those with UC without depression or anxiety (UCND/UCNA) (118). Correspondingly, the researchers also found an obvious increase in the abundance of the *Enterococcus* genus and a reduction in, among others, *Bifidobacterium*, *Roseburia*, Lachnospiraceae/*Lachnospira*, and *Ruminococcus* in IBD patients (119, 128, 129). From this point of view, these studies also suggested that psychological stress aggravating IBD might be related to the amplification of intestinal pathogens and the decrease in the number of beneficial bacteria. However, because the above-mentioned research comprise cross-sectional observational studies of gut microbiota, it remains to be clarified whether the observed changes in the gut microbiome are a cause or a consequence of IBD and psychological disorders, and a comprehensive dissection of the interactions between the microbiota and the host is needed. It is also necessary to use germ-free animal models to verify the validity of the conclusions. A recent Fecal microbiota transplantation (FMT) study has partially solved these problems (126). The results of this study showed that fecal transplantation in IBD patients with depression (IBD/D^+^-F) caused more severe IBD-like colitis in specific pathogen-free (SPF) recipient mice than in IBD patients without depression (IBD/D^−^-F). Additionally, it should be noted that the Enterococcaceae population was higher in IBD/D^+^-F than in IBD/D^−^-F (126). Unfortunately, the study used the SPF mice model rather than the germ-free mice model. Nevertheless, the above-discussed studies suggest that the alterations of the intestinal microbiota might be not only a vital factor in the appearance of psychological symptoms but also a target of future microbiota-modulating therapeutic strategies targeting at IBD patients or IBD patients with psychological comorbidities.

In addition to the intestinal microbiota itself, the microbial metabolites also participate in the pathogenesis of colitis. Short-chain fatty acids (SCFAs), one of the important metabolites of intestinal flora, play a major role in several physiological processes, including the maintenance of the intestinal barrier, the inhibition of opportunistic intestinal pathogen colonization, and the regulation of the Th17/RORγt^+^ Treg cell balance (130, 131). A recent study of the animal model revealed that CUMS causes a significant decline in the intestinal *Prevotella*-9 and *Alloprevotella* abundance (120). Furthermore, clinical studies also found that the relative abundance of *Roseburia*, the butyrate-producing bacterial strain, was negatively correlated with depression in CD (127, 132). As mentioned above, compared to UCND/UCNA, UCD/UCA had less *Prevotella*-9 and *Lachnospira* (118). Microbial dysbiosis and low abundance of butyrate-producing bacteria, including *Prevotella*-9 and *Lachnospira*, reduces butyrate levels in the intestine, lowers intestinal epithelial tight junction protein expression, and decreases the number of goblet cells, resulting in damage to the barrier function of the mucosal layer and aggravating the colonic inflammatory response (118, 120). Additionally, the levels of a few metabolites, such as 2’-deoxy-D-ribose and L-pipecolic acid, which were enriched in UCND/UCNA, were decreased, accompanied by a reduction in immunoglobulin proteins in UCD/UCA, implying that the two beneficial metabolites might play a significant role in the host immune response of UCD/UCA. Particularly, the prophylactic administration of these metabolites could significantly alleviate the depressive-like behaviors in mice with colitis and decrease central and peripheral circulating inflammatory cytokine levels (118). The above studies have strongly confirmed that stress can induce alterations to the fecal microbiota and metabolites, further aggravating intestinal inflammation. Interestingly, the modulation of the intestinal microbiota changes stress responses, further emphasizing the bidirectionality of the brain–gut axis. FMT has become an attractive therapeutic strategy (133). It has shown that the severity of anxiety, depression and obsession in IBD patients decreased after FMT (134). In a stress model that mimics social isolation, altering the intestinal flora with the administration of rifaximin could reduce stress responses and brain function deterioration (135). In another repeated psychosocial stress model, SCFAs supplementation alleviates increase in intestinal permeability (136). Such findings were complemented by those of studies assessing the protective effect of probiotics during stress. For example, it has been demonstrated that treatment with probiotics (i.e., *Lactobacillus casei* [LcS]) relieved stress-induced abdominal dysfunction (137). Probiotic supplements also may alleviate stress-induced colitis by suppressing gut bacterial LPS and subsequently inhibiting the intestinal inflammatory response (138).

## IBD leads to anxiety- and depression-like behavioral comorbidities

Studies have shown that anxiety/depression-like behavior, as -comorbidity, is likely to evolve into one of the main features of IBD (139–141). Moreover, long-term follow-up of patients found that patients who were clinically active at baseline but had no symptoms of anxiety or depression at the start of the study had a significantly higher risk of developing new-onset anxiety or depression symptoms (142). However, the pathologic mechanisms by which intestinal inflammation induces cerebral structural changes and, thus, lead to behavioral comorbidities remain unclear. Based on gathered evidence, we proposed the potential mechanism by which IBD leads to anxiety/depression (**Figure 2**).

It has been shown that circulating pro-inflammatory mediators in IBD patients might be the main culprits promoting the development of CNS inflammation. Consequently, increasing evidence indicates that mood disorders are associated with altered inflammatory statuses in the CNS (143–145). In some studies involving the use of experimental colitis animal models, peripheral inflammation elevated the levels of proinflammatory cytokines such as TNF-α, IL-1β, and IL-6 in the hippocampus, cerebral cortex, and hypothalamus (146–153). Furthermore, researchers have also found the overexpression of TLR-4, phosphorylated-NF-κB p65, myeloid differentiation primary response 88 (Myd88), and NOD-like receptor protein 3 (NLRP3) in the hippocampi of animals in IBD groups compared with control animals, indicating the activation of the TLR4/NF-κB inflammatory pathway and NLRP3 inflammasomes in the CNS (148, 153, 154). That evidence suggests that colitis could lead to CNS inflammation, which culminates in the occurrence of abnormal behavior in animals. Additionally, peripheral inflammation also significantly increased inducible NO synthase (iNOS) and nitrite levels in the hippocampus and cerebral cortex, contributing to the occurrence of anxiety- and depression-like behavioral comorbidities (151, 154, 155). For example, compared with those in the control group, mice with colitis showed longer-lasting immobility in the forced swimming test (FST), and higher expression levels of pro-inflammatory mediators (including TNF-α), iNOS expression, and nitrite content in the hippocampus. However, when NOS inhibitors were used to prevent iNOS and nitrite production, mice with colitis had a shorter rest time in FST, lower TNF-α and nitrite levels in the hippocampus, and reduced inflammatory injury in the hippocampal region (155).

Hence, the crucial mechanism by which peripheral inflammation contributes to the progression of several CNS disorders remains unknown. Other studies demonstrated that colitis can increase the amounts of bacteria-derived toxic byproducts (LPS), pro-inflammatory cytokines (including TNF-α, IL-1β and IL-6), and gut leak (126, 153, 156). Moreover, the expression levels of tight junction proteins, including ZO-1, occludin, and zonulin, were significantly decreased in the cerebral tissues of experimental animals with colitis (153, 156, 157). These findings have confirmed that colitis can cause endothelial damage to the BBB. Furthermore, a broken BBB is beneficial in translocating circulating proinflammatory mediators into cerebral tissue, leading to the inflammatory response in the CNS and affecting neuroglial networks and activated microglial cells that further exacerbate the inflammatory response in the CNS (145). Additionally, activated microglial cells can impair the proliferation and differentiation of hippocampal progenitor cells by producing serotonin-depleting enzymes and mediators, and consequently, promoting neurodegeneration, which is associated with the pathophysiology of depression-like behaviors (158, 159).

More recently, new mechanisms leading to these behavioral comorbidities in IBD have been proposed. Carloni et al. (160) described a vascular barrier existing in the brain choroid plexus (PVB), which is able to respond to intestinal inflammation through bacteria-derived LPS. An *in vitro* experimental model of choroid plexus endothelial cells has shown that PVB closure is associated with short-term memory and mental deficits. Speculation that the dysfunction of the gut-brain vascular axis might be a factor worthy of attention leading to IBD-related psychiatric comorbidities. Additionally, Chen et al. (161) reported that IBD-related psychological disorders could be related to the activation of the kynurenine pathway (KP) due to chronic inflammation. After the activation of KP, excessive neurotoxic quinolinic acid and less neuroprotective kynerunic acid were produced in cerebral tissue, ultimately resulting in inflammation-induced depression.

Understanding the mechanisms of how IBD leads to psychiatric comorbidities and exploring the causal relationship between these comorbidities and IBD are important for the prevention, treatment, and prognosis of IBD. Although the current studies are mostly limited to animal models, many of the findings provide valuable clues to clarify the potential link between IBD and anxiety- and depression-like behaviors in humans. With the development of functional brain imaging technology that can monitor the changes in brain signaling and morphology, the mechanism of the brain–gut bidirectional communications and novel avenues for intervention can be further clarified.

## Application of neuroimaging studies in evaluating the possible neuromechanisms of IBD

Brain–gut interactions may be able to explain the increased prevalence of psychiatric symptoms in IBD, however, studies on the pathological changes in the brain caused by IBD remain equivocal. In recent years, functional brain imaging mainly including positron emission tomography (PET) and functional magnetic resonance imaging (fMRI), has enabled *in vivo* analyses of the interactions and neurological mechanisms between the brain and digestive tract, advancing the scientific understanding of this relationship. Compared with PET, fMRI is non-invasive, safer and easier to operate. Therefore, it is more widely used to image changes in brain function in IBD (162–165).

Agostini et al. (166) used fMRI to investigate the neural emotional changes in UC patients in remission *versus* healthy controls. They found that patients with UC manifest a decrease in sensitivity to positive emotions of joy and well-being. Because all patients enrolled in their study were in remission, the emotional dysfunction was not strictly associated with current inflammatory activity. Nevertheless, their findings represent a preliminary but interesting result that might reveal novel avenues for the study of the brain–gut axis and for understanding the relationship between mood and intestinal chronic inflammation (166). More recently, research has found that changes in gray matter volume (GMV) in regions, such as the insula, pregenual anterior cingulated cortex, thalamus, amygdala, supplementary motor area, periaqueductal gray, hypothalamus, and precentral gyrus, which were involved in visceral sensory pathways, were more commonly observed in UC patients using structural MRI than in healthy controls. Meanwhile, compared to patients with UC in remission, those in the active phase have a much greater chance of developing GMV changes, thus, the researchers claimed that changes in the brain could be partially connected with the clinical stage in patients with UC (167). Interestingly, multimodal brain MRI analysis has shown that behavioral symptoms are strongly associated with structural and functional changes in deep gray matter that modulate emotional, cognitive, and stress responses in IBD patients. Compared to healthy controls, the patients with UC or CD exhibited the increased volumes of amygdale and hypothalamus, the neurodegeneration of putamen and pallidum, and significantly increased activity and functional connectivity (FC) in cognitive and emotional processing brain regions, including the hypothalamus, basal ganglia, and limbic system. Particularly, an increase in the volume of the thalamus can reflect an exacerbated inflammatory state in the brain. Meanwhile, the researchers also found that the hippocampal nerve activity is increased with IBD in the active phase compared with those in remission (168). Additionally, a dynamic brain functional connectome analysis using fMRI has revealed medial prefrontal cortex dysfunction, which is involved in the deterioration of depression and anxiety in patients with UC (169). Those studies provide a promising perspective and convincing neuroimaging evidence of potential neuromechanisms of UC.

fMRI can also be used to provide additional information about changes in the brain structure of CD patients. CD patients display altered grey matter structures and grey matter structural connectome, which just to a certain extent gives the reason why these patients showed higher levels of anxiety and depression (163, 170). Stress-induced hyperactivity has been found in particular brain regions of CD patients, including the amygdala and midcingulate cortex (171, 172). These alterations might represent a disruption of the brain–gut axis connection that could predispose IBD patients to psychological comorbidities and need to be considered during treatment. It is worthnoting that recent studies have focused on brain functional alterations in CD patients during the resting-state (173–177). Compared to healthy controls, fMRI analysis demonstrated that the CD patients during the resting-state of brain show the altered FC of pre-frontal cortex by increasing FC between the cognitive control and salience network and decreasing within the default mode network (174). The findings of another fMRI analysis explored the aberrant FC of the amygdala with the multiple regions, including and insula, parahippocampus, as well as anterior middle cingulate cortex/dorsal anterior cingulate cortex (173). Additonally, a recent meta-analysis study for the existing neuroimaging data indicated that, compared to healthy controls, patients with CD during the resting-state of brain had decreased FC in the paracentral lobule and cingulated gyrus as well as reduced GMV in the medial frontal gyrus (175). These brain functional alterations suggested that the aberrant FC may be associated with high sensitivity in negative emotion and reduced inhibition on processing of visceral pain and sensation in CD patients during the resting-state of brain. Excitingly, it has indicated that the improved cognitive deficits in major depressive disorder were linked to alterations in limbic (amygdala) function following anti-TNF-α treatment (178). Thus, the urgent issues should investigate not only potential network alterations during different CD states especially the period of active disease and even after treatment but also combine various neuroimaging and psychometric approaches to clarify how these respective alterations are related to each other.

Although a growing number of neuroimaging studies have shown significant neurological changes in IBD, small sample sizes and cross-sectional studies might undermine the credibility of these results. Furthermore, since UC and CD respectively share distinct pathological features, it is unclear whether such distinct pathological features lead to different neuroanatomical changes. Therefore, future studies should examine more patients with CD and UC to determine whether there are significant neurological differences between the two specific diseases. Additionally, to determine whether brain structural alterations correlate with disease activity, researchers should include patients with different disease states to investigate separately in a longitudinal design.

## Psychotherapy and antidepressants for patients with IBD

Recent research findings have suggested that psychological interventions can improve the treatment effect of gastrointestinal diseases, which will improve the QoL of patients (179–184). Among a variety of psychological interventions, cognitive behavioral therapy (CBT) is recognized as the most effective psychotherapy for managing IBD, which can reduce the rate of psychological disorder and improve QoL in IBD patients (185–189). Furthermore, in a benchmarking study, CBT not only significantly reduced scores of anxiety and low mood and significantly increased sores of QoL but also decreased disease activity in the specific group of IBD patients who also had anxiety and low mood (186). Similarly, treatment with CBT has been associated with a significantly greater improvement in the depressive severity in the overall sample of young people with depression and CD and correlated with a significantly greater improvement in the pediatric CD activity in the subset with active IBD (190). Additionally, a randomized trial indicated that multicomponent CBT (MCBT) is highly effective in alleviating low mood and improving QoL in stress-prone IBD patients. Noticeably, this program could effectively reduce the relapse rate of patients; however, no differences in disease activity indexes were found (191). As far as the current study is concerned, a recent systematic review has claimed that the positive effects of CBT on improving the psychological status of IBD patients are not long-lasting, and there is insufficient evidence that CBT improves disease activity or reduces inflammation levels (192).

Mindfulness-based interventions (MBIs), which primarily manifest as attention to one’s physical sensations, thoughts, and emotions, have also been shown to have a positive impact on relieving stress levels and improving QoL in IBD patients (193–196). For example, patients with inactive UC demonstrated significantly lower stress and higher QoL after mindfulness-based stress reduction treatment (197). Recently, a randomized controlled trial has also demonstrated that MBIs had a positive impact on alleviating psychological disorders, fatigue, and improving the QoL of patients with CD. Particularly, CD patients with severe baseline psychological symptoms were the greatest beneficiaries of MBIs (198). Additionally, some studies have indicated that MBIs likely represented a promising intervention to reduce inflammation in IBD patients because MBIs could effectively decrease the levels of mucosal inflammatory biomarkers, including IL-6, fecal calprotectin, and CRP levels (194, 196, 198). Thus far, limited support is available for the effects of MBI on disease-related and physiologic outcomes. Therefore, future studies should pay more attention to the clarification of the physiological effects of MBIs on IBD, and find reliable physiological indicators to evaluate its effects.

Currently, various types of psychotherapy interventions–, such as gut-directed hypnotherapy, participation in the breath-body-mind workshop, advanced combination treatment, specialized educational and psychological counseling, and acceptance and commitment therapy–, are available (184, 199–202). The effectiveness of these psychological interventions for managing IBD is also being studied; however, there is still a paucity of trial evidence of efficacy for most of them. For example, it is not certain that hypnotherapy can relieve IBD symptoms and prolong the remission period during UC (203, 204). Although the effect of psychological therapy on IBD is controversial, 30%–50% of- IBD patients with reduced QoL are expected to receive complementary psychological interventions (205, 206). Additionally, it has been demonstrated that patients with active disease, especially during a flare, have higher anxiety/depression and lower QoL scores and appear to benefit more from psychotherapy than those in remission (207, 208). Therefore, more high-quality studies are needed to design personalized psychotherapy whose early intervention would improve coping strategies and QoL scores for IBD patients in the future.

Accompanied by psychotherapy, antidepressants have been considered as potential adjunctive treatments for IBD with psychological comorbidities. Firstly, some studies have indicated that antidepressants such as Tianeptine and Duloxetine have positive influence on the improvement of severity of psychological and physical symptoms of patients with IBD (209). Furthermore, it has shown that antidepressants can help mitigate coexisting functional gastrointestinal symptoms in IBD. Some studies have demonstrated that Tricyclic antidepressants (TCAs) have efficacy in treating residual gastrointestinal symptoms and slowing colonic transit in IBD patients (210, 211). Excitingly, antidepressants may also have a positive effect on the inflammatory state of IBD. In animal models of IBD, both desipramine and fluoxetine significantly attenuated colonic pathological damage and lowered serum concentrations of TNF-α and IL-1β (212, 213). Several human trials have observed that antidepressant fluoxetine show modest effects on immune functions (214), and antidepressants tianeptine and duloxetine can significantly improve the disease activity (209). Additionally, antidepressants might relieve chronic pain during remission and improve impaired sleep quality in patients with IBD (215, 216).

Antidepressants may be beneficial in the treatment of IBD, however, the evidence supporting this conclusion is insufficient, the quality of the evidence is very low to moderate and requires further investigation. Limited preliminary studies has shown the potential of antidepressants for IBD, which needs to be validated by a large number of well-designed clinical randomizedcontrolledtrial (RCT) trials with a longer follow-up in the future. Although no major adverse events have been reported with antidepressants for IBD, there is a potential risk of gastrointestinal-specific adverse effects, especially when more than one antidepressant is used (214, 217). At present, it is not clear which class or specific drug might be the most effective for patients with IBD alone or for those with psychological comorbidities (218). Despite the studies in this area is still deficient, accumulating evidence suggests that antidepressant treatments have the potential to mitigate the risk of IBD, ameliorate mental health and QoL in some IBD patients, and alter the natural history of the disease (219–221).

## Conclusion

Chronic psychological stress accelerates IBD progression by affecting the neuroendocrine-immune regulatory network and disturbing intestinal mucosal immunity and microbiome homeostasis. Of note, IBD might also lead to anxiety- and depression-like behavioral comorbidities by over-activating brain immune regulation. Thus, there is clear bidirectionality of the brain–gut axis in IBD and psychological stress. Brain–gut interactions could explain the increased prevalence of psychological disorders in IBD patients; however, the pathological changes in the cerebral structures in IBD patients cannot be ignored. Functional brain imaging is a valuable method of investigating the possible neuromechanisms of IBD underlying visceral disturbances. Current findings have suggested that IBD might have a negative impact on cerebral structure networks that manifest as widespread neuroanatomical changes associated with stress. A deeper study of brain structural networks in IBD patients will help us develop adjuvant therapies to treat extraintestinal comorbid conditions, such as depression or anxiety.

Although we are now starting to understand the underlying mechanism of the link between psychological stress and IBD, we do not know how often psychological disorders co-occur with IBD, nor do we know the causal relationship between them. Despite the current study is limited, fortunately microbiome manipulations by using fecal microbiota transplantation or administration of microbial metabolites or probiotics can target specific pathways in IBD pathogenesis and promise to play a crucial role in the treatment of IBD and its concomitant psychiatric disorders. The development of microbiota-targeted treatment strategies will change the disease course and improve the mental health and QoL of patients with IBD (133, 134, 136, 138, 222, 223). Futhermore, psychotherapy and antidepressants have also become promising areas. Until now there is not enough evidence to prove that psychotherapy and antidepressants are effective, however, the early intervention of the designed personalized psychotherapy and appropriate use of antidepressants will be expected to improve coping strategies and QoL scores for IBD patients in the future. Additonally, current studies have also largely relied on murine models, and validation using other advanced preclinical IBD models and IBD patients is needed. Comprehensive clinical studies with large sample sizes combined with more high-quality research are needed to create personalized treatment strategies for IBD patients. In conclusion, there is increasing evidence that the brain–gut axis regulates disease progression.Translating these discoveries into psychoneuroimmunology will be essential for clarifying the pathological mechanism of IBD with psychological comorbidities and designing clinical treatment protocols.

## Author contributions

LG and SuL were involved in writing the manuscript. SL and JY performed the majority of literature retrieval work. GH designed the tables and figures. CX and WS were responsible for proposing the framework and revising the manuscript. All authors contributed to the article and approved the submitted version.

## Funding

This work was supported by the National Natural Science Foundation of China (82271803), the Natural Science Foundation of Shandong Province (ZR2019MH123), the Academic Promotion Programme of Shandong First Medical University (2019QL007, YS22-0001817), the Open Project of Shandong Provincial Key Laboratory of Animal Biotechnology and Disease Control and Prevention (ABDC-201901), and the Shandong Provincial Integrated Traditional Chinese and Western Medicine Special Disease Prevention Project (YXH2019ZXY003).

## Acknowledgments

The manuscript has been reviewed, and the syntax and grammar have been edited by Charlesworth Author Services. Additionally, we thank Shanghai Sensichip Infotech Co. Ltd, Shanghai, China, for their mapping services.

## Conflict of interest

The authors declare that the research was conducted in the absence of any commercial or financial relationships that could be construed as a potential conflict of interest.

## Publisher’s note

All claims expressed in this article are solely those of the authors and do not necessarily represent those of their affiliated organizations, or those of the publisher, the editors and the reviewers. Any product that may be evaluated in this article, or claim that may be made by its manufacturer, is not guaranteed or endorsed by the publisher.
